# Crystal structure of *trans*-cyclo­hexane-1,2-di­ammonium chromate(VI) from synchrotron X-ray diffraction data

**DOI:** 10.1107/S2056989016019009

**Published:** 2016-11-30

**Authors:** Dohyun Moon, Jong-Ha Choi

**Affiliations:** a Pohang Accelerator Laboratory, POSTECH, Pohang 37673, Republic of Korea; b Department of Chemistry, Andong National University, Andong 36729, Republic of Korea

**Keywords:** crystal structure, *trans*-cyclo­hexane-1,2-di­ammonium, chromate(VI), hydrogen bonding, synchrotron radiation, hybrid compound

## Abstract

The structure of the title hybrid compound, (C_6_H_16_N_2_)[CrO_4_], has been determined from synchrotron data. The organic cation adopts a chair conformation. The inorganic CrO_4_^2−^ anion is slightly distorted owing to its involvement in N—H⋯O hydrogen-bonding inter­actions with neighbouring *trans*-cyclo­hexane-1,2-di­ammonium cations, whereby the two Cr—O bonds to the O atoms acting as acceptor atoms for two hydrogen bonds are slightly longer than the other two Cr—O bonds for which only one acceptor inter­action per O atom is observed. In the crystal, cations and anions are packed into layers parallel to (001), held together through the aforementioned N—H⋯O hydrogen bonds.

## Abstract 2

In the title compound, (C_6_H_16_N_2_)[CrO_4_], the *trans*-cyclo­hexane-1,2-di­ammonium cations and chromate anions are connected through N—H⋯O hydrogen bonds. The tetra­hedral CrO_4_^2−^ anion is slightly distorted due to the influence of the hydrogen bonds.

## Chemical context   

Organic–inorganic hybrid compounds are of inter­est because of the possibility of their forming extended networks through versatile hydrogen bonds (Mkaouar *et al.*, 2016 ▸). The amine *trans*-1,2-cyclo­hexa­nedi­amine (chxn), C_6_H_14_N_2_, is strongly basic and readily captures two protons to form a dication, (C_6_H_16_N_2_)^2+^. Crystal structures of this amine or the dication have been determined for *trans*-1,2-cyclo­hexa­nedi­amine hydro­bromide (Morse & Chesick, 1976 ▸), *trans*-cyclo­hexane-1,2-di­ammonium dichloride (Farrugia *et al.*, 2001 ▸) and *trans*-cyclo­hexane-1,2-di­ammonium bis­(3′-nitro-*trans*-cinnamate) (Hosomi *et al.*, 2000 ▸). With respect to complex inorganic anions of the types ZnCl_4_^2−^, CrO_4_^2−^ or Cr_2_O_7_^2−^, the crystal structures of hybrid compounds with organic ammonium cations have been determined for propane-1,3-di­ammonium tetra­chlorido­zincate (Kallel *et al.*, 1980 ▸), propane-1,3-di­ammonium dichromate(VI) (Trabelsi *et al.*, 2012 ▸) and propane-1,2-di­ammonium chromate(VI) (Trabelsi *et al.*, 2014 ▸). However, a combination of *trans*-cyclo­hexane-1,2-di­ammonium and CrO_4_^2−^ has not been reported. In this communication, we present details on the preparation of the new organic chromate(VI), (C_6_H_16_N_2_)[CrO_4_], (I) and its structural characterization by synchrotron single-crystal X-ray diffraction.

## Structural commentary   

**Figure 1:**
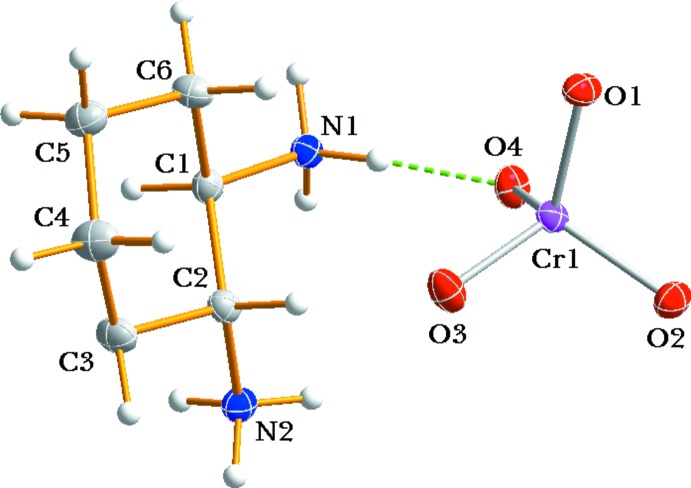
The mol­ecular structures of the organic cation and the inorganic anion in (I), drawn with displacement ellipsoids at the 30% probability level. The dashed line represents a hydrogen-bonding inter­action.

Fig. 1 ▸ shows an ellipsoid plot of the mol­ecular components of (I). The organic di­ammonium cation adopts a stable chair conformation with respect to the cyclo­hexane ring. The C—C and N—C distances range from 1.506 (5) to 1.525 (4) Å and from 1.492 (3) to 1.493 (3) Å, respectively; the range of N—C—C and C—C—C angles is 108.3 (2) to 113.7 (2)° and 109.2 (2) to 112.0 (3)°, respectively.
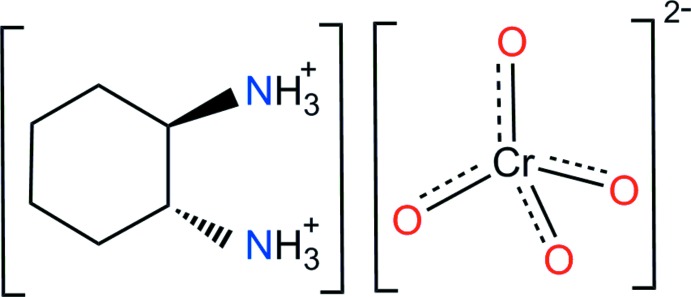


The bond lengths and angles are very similar than in the structure of the bis­(3′-nitro-*trans*-cinnamate) compound with the same cation (Hosomi *et al.*, 2000 ▸). The cyclo­hexane ring C—C bond lengths and angles and the torsion angles involving the C and N atoms are in essential agreement with the values obtained for [Cr(chxn)_3_](ZnCl_4_)Cl·3H_2_O (Moon & Choi, 2016 ▸). The Cr^VI^ atom in the CrO_4_^2−^ anion has the characteristic tetra­hedral coordination environment of four O atoms, with Cr—O bond lengths ranging from 1.628 (2) to 1.6654 (19) Å and O—Cr—O angles ranging from 108.30 (10)–111.43 (11)° (Table 1 ▸). The distortion from ideal values is due to the influence of hydrogen bonding. For O atoms that are acceptor atoms of two hydrogen bonds (O1 and O4), the Cr—O bond lengths are slightly longer than those of the other two O atoms (O2 and O3) which are each involved in only one hydrogen-bonding inter­action.

## Supra­molecular features   

**Figure 2:**
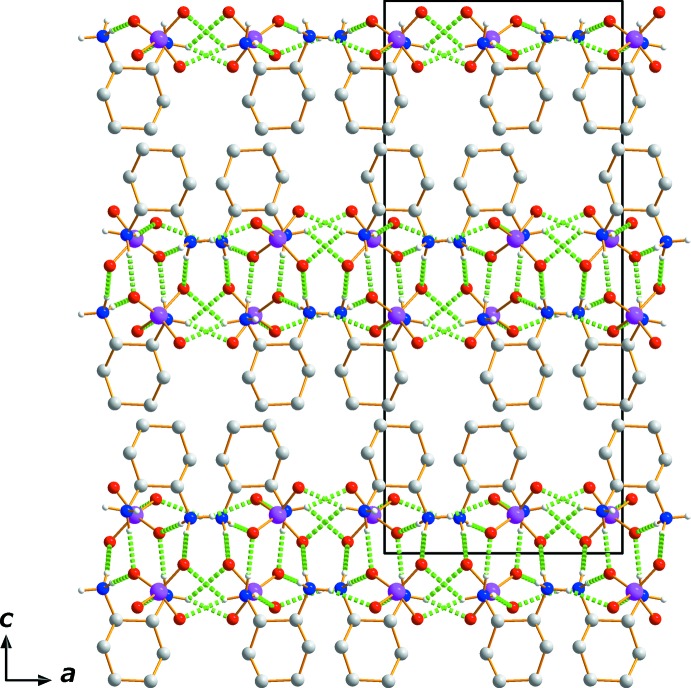
The crystal packing in (I), viewed along [010]. Hydrogen-bonding inter­actions are indicated by dashed lines.

In the crystal structure, the cations and anions are arranged in layers parallel to (001). The ammonium group is directed towards the anion, hence causing polar and non-polar sections in the crystal structure, alternating along [001]. As mentioned above, each of the O atoms is involved in N—H⋯O hydrogen bonds that hold the polar (001) sheets together (Fig. 2 ▸, Table 2 ▸).

## Database survey   

A search of the Cambridge Structural Database (Version 5.37, Feb 2016 with three updates; Groom *et al.*, 2016 ▸) indicates a total of 31 hits for compounds containing the cyclo­hexa­nedi­ammonium cation (C_6_H_16_N_2_)^2+^.

## Synthesis and crystallization   

Compound (I) was prepared by dissolving 5 mmol of chromium trioxide (0.50 g, Sigma–Aldrich) and 0.5 mmol of *trans*-1,2-cyclo­hexa­nedi­amine (0.6 mL, Sigma-Aldrich) in 40 mL of distilled water with a molar ratio of 1:1. The mixture was stirred for 30 minutes and the resulting solution was allowed to stand at room temperature for one day to give plate-like yellow crystals suitable for X-ray structural analysis.

## Refinement   

Crystal data, data collection and structure refinement details are summarized in Table 3 ▸. All H atoms were placed in geometrically idealized positions and constrained to ride on their parent atoms, with C—H = 0.99-1.00 Å and N—H = 0.91 Å, and with *U*_iso_(H) values of 1.2 or 1.5*U*_eq_ of the parent atoms.

## Supplementary Material

Crystal structure: contains datablock(s) I. DOI: 10.1107/S2056989016019009/wm5343sup1.cif

Structure factors: contains datablock(s) I. DOI: 10.1107/S2056989016019009/wm5343Isup2.hkl

CCDC reference: 1519508

Additional supporting information: crystallographic information; 3D view; checkCIF report

## Figures and Tables

**Table 1:** Selected geometric parameters (Å, °)

Cr1—O3	1.628 (2)	Cr1—O1	1.6584 (19)
Cr1—O2	1.6394 (19)	Cr1—O4	1.6654 (19)
			
O3—Cr1—O2	108.60 (11)	O3—Cr1—O4	109.76 (10)
O3—Cr1—O1	111.43 (11)	O2—Cr1—O4	108.30 (10)
O2—Cr1—O1	109.72 (10)	O1—Cr1—O4	108.97 (10)

**Table 2:** Hydrogen-bond geometry (Å, °)

*D*—H⋯*A*	*D*—H	H⋯*A*	*D*⋯*A*	*D*—H⋯*A*
N1—H1*N*1⋯O2^i^	0.91	1.99	2.896 (3)	172
N1—H3*N*1⋯O1^ii^	0.91	2.00	2.884 (3)	164
N1—H2*N*1⋯O4	0.91	1.81	2.713 (3)	175
N2—H1*N*2⋯O4^i^	0.91	1.87	2.771 (3)	169
N2—H3*N*2⋯O2^iii^	0.91	2.56	3.104 (3)	119
N2—H3*N*2⋯O3^iii^	0.91	2.04	2.927 (3)	166
N2—H2*N*2⋯O1^iv^	0.91	1.86	2.748 (3)	165

Symmetry codes: (i) 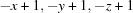; (ii) 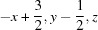; (iii) 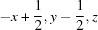; (iv) 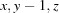.

**Table 3:** Experimental details

Crystal data	
Chemical formula	(C_6_H_16_N_2_)[CrO_4_]
*M*_r_	232.21
Crystal system, space group	Orthorhombic, *P* *b* *c* *a*
Temperature (K)	173
*a*, *b*, *c* (Å)	9.910 (2), 8.3730 (17), 22.999 (5)
*V* (Å^3^)	1908.4 (7)
*Z*	8
Radiation type	Synchrotron, λ = 0.650 Å
μ (mm^−1^)	0.92
Crystal size (mm)	0.10 × 0.09 × 0.01

Data collection	
Diffractometer	ADSC Q210 CCD area detector
Absorption correction	Empirical (using intensity measurements) (*HKL3000sm *SCALEPACK**; Otwinowski & Minor, 1997 ▸)
*T*_min_, *T*_max_	0.794, 1.000
No. of measured, independent and observed [*I* > 2σ(*I*)] reflections	16426, 2383, 1749
*R*_int_	0.069
(sin θ/λ)_max_ (Å^−1^)	0.674

Refinement	
*R*[*F* ^2^ > 2σ(*F* ^2^)], *wR*(*F* ^2^), *S*	0.055, 0.160, 0.99
No. of reflections	2383
No. of parameters	121
H-atom treatment	H-atom parameters constrained
Δρ_max_, Δρ_min_ (e Å^−3^)	0.95, −1.53

Computer programs: *PAL BL2D-SMDC* (Shin *et al.*, 2016 ▸), *HKL3000sm* (Otwinowski & Minor, 1997 ▸), *SHELXT2014* (Sheldrick, 2015*a* ▸), *SHELXL2014* (Sheldrick, 2015*b* ▸), *DIAMOND* (Putz & Brandenburg, 2014 ▸) and *publCIF* (Westrip, 2010 ▸).

## supplementary crystallographic information

### Crystal data

**Table:**

(C_6_H_16_N_2_)[CrO_4_]	*D*_x_ = 1.616 Mg m^−^^3^
*M**_r_* = 232.21	Synchrotron radiation, λ = 0.650 Å
Orthorhombic, *P**b**c**a*	Cell parameters from 49521 reflections
*a* = 9.910 (2) Å	θ = 0.4–33.4°
*b* = 8.3730 (17) Å	µ = 0.92 mm^−^^1^
*c* = 22.999 (5) Å	*T* = 173 K
*V* = 1908.4 (7) Å^3^	Plate, yellow
*Z* = 8	0.10 × 0.09 × 0.01 mm
*F*(000) = 976	

### Data collection

**Table:**

ADSC Q210 CCD area detector diffractometer	1749 reflections with *I* > 2σ(*I*)
Radiation source: PLSII 2D bending magnet	*R*_int_ = 0.069
ω scan	θ_max_ = 26.0°, θ_min_ = 2.5°
Absorption correction: empirical (using intensity measurements) (*HKL3000sm Scalepack*; Otwinowski & Minor, 1997)	*h* = −12→12
*T*_min_ = 0.794, *T*_max_ = 1.000	*k* = −11→11
16426 measured reflections	*l* = −31→31
2383 independent reflections	

### Refinement

**Table:**

Refinement on *F*^2^	Hydrogen site location: inferred from neighbouring sites
Least-squares matrix: full	H-atom parameters constrained
*R*[*F*^2^ > 2σ(*F*^2^)] = 0.055	*w* = 1/[σ^2^(*F*_o_^2^) + (0.116*P*)^2^] where *P* = (*F*_o_^2^ + 2*F*_c_^2^)/3
*wR*(*F*^2^) = 0.160	(Δ/σ)_max_ < 0.001
*S* = 0.99	Δρ_max_ = 0.95 e Å^−^^3^
2383 reflections	Δρ_min_ = −1.53 e Å^−^^3^
121 parameters	Extinction correction: SHELXL2014 (Sheldrick, 2015b), Fc^*^=kFc[1+0.001xFc^2^λ^3^/sin(2θ)]^-1/4^
0 restraints	Extinction coefficient: 0.017 (3)

### Special details

**Table:**

Geometry. All esds (except the esd in the dihedral angle between two l.s. planes) are estimated using the full covariance matrix. The cell esds are taken into account individually in the estimation of esds in distances, angles and torsion angles; correlations between esds in cell parameters are only used when they are defined by crystal symmetry. An approximate (isotropic) treatment of cell esds is used for estimating esds involving l.s. planes.

### Fractional atomic coordinates and isotropic or equivalent isotropic displacement parameters (Å^2^)

**Table:**

	*x*	*y*	*z*	*U*_iso_*/*U*_eq_	
N1	0.6842 (2)	0.3903 (3)	0.43614 (9)	0.0245 (5)	
H1N1	0.6673	0.3156	0.4639	0.037*	
H2N1	0.6444	0.4842	0.4463	0.037*	
H3N1	0.7749	0.4049	0.4328	0.037*	
N2	0.4159 (3)	0.2284 (3)	0.42353 (10)	0.0279 (5)	
H1N2	0.4273	0.2758	0.4588	0.042*	
H2N2	0.4575	0.1315	0.4235	0.042*	
H3N2	0.3262	0.2151	0.4165	0.042*	
C1	0.6285 (3)	0.3349 (3)	0.37930 (11)	0.0249 (6)	
H1	0.6622	0.2241	0.3721	0.030*	
C2	0.4758 (3)	0.3313 (3)	0.37730 (12)	0.0247 (5)	
H2	0.4413	0.4427	0.3823	0.030*	
C3	0.4292 (3)	0.2686 (4)	0.31840 (13)	0.0342 (7)	
H3A	0.4600	0.1569	0.3137	0.041*	
H3B	0.3293	0.2688	0.3171	0.041*	
C4	0.4833 (3)	0.3687 (4)	0.26844 (14)	0.0410 (8)	
H4A	0.4570	0.3192	0.2310	0.049*	
H4B	0.4430	0.4768	0.2701	0.049*	
C5	0.6347 (3)	0.3820 (4)	0.27138 (13)	0.0357 (7)	
H5A	0.6665	0.4563	0.2408	0.043*	
H5B	0.6754	0.2760	0.2638	0.043*	
C6	0.6808 (3)	0.4421 (4)	0.33063 (12)	0.0318 (6)	
H6A	0.6475	0.5523	0.3366	0.038*	
H6B	0.7807	0.4447	0.3318	0.038*	
Cr1	0.44912 (4)	0.79269 (5)	0.43056 (2)	0.0227 (2)	
O1	0.53896 (18)	0.9403 (2)	0.40203 (9)	0.0308 (5)	
O2	0.34508 (19)	0.8641 (2)	0.47950 (9)	0.0330 (5)	
O3	0.3616 (2)	0.6998 (2)	0.38110 (10)	0.0362 (5)	
O4	0.55315 (18)	0.6648 (2)	0.46324 (9)	0.0311 (5)	

### Atomic displacement parameters (Å^2^)

**Table:**

	*U*^11^	*U*^22^	*U*^33^	*U*^12^	*U*^13^	*U*^23^
N1	0.0243 (12)	0.0215 (11)	0.0278 (11)	0.0015 (9)	−0.0012 (8)	0.0002 (8)
N2	0.0267 (13)	0.0252 (12)	0.0316 (13)	0.0024 (10)	0.0025 (9)	0.0012 (9)
C1	0.0249 (14)	0.0217 (12)	0.0279 (13)	0.0026 (10)	0.0002 (10)	−0.0011 (10)
C2	0.0257 (14)	0.0195 (12)	0.0289 (13)	0.0000 (10)	−0.0010 (10)	0.0009 (10)
C3	0.0314 (18)	0.0406 (16)	0.0307 (15)	−0.0072 (12)	−0.0047 (11)	−0.0009 (13)
C4	0.0376 (18)	0.055 (2)	0.0309 (15)	−0.0053 (16)	−0.0050 (12)	0.0073 (14)
C5	0.0317 (16)	0.0437 (17)	0.0315 (15)	−0.0018 (13)	0.0024 (11)	0.0029 (12)
C6	0.0298 (16)	0.0322 (14)	0.0334 (15)	−0.0062 (12)	0.0000 (11)	0.0044 (11)
Cr1	0.0223 (3)	0.0167 (3)	0.0290 (3)	0.00140 (14)	−0.00086 (15)	0.00134 (14)
O1	0.0285 (11)	0.0233 (10)	0.0405 (12)	−0.0002 (8)	0.0062 (8)	0.0049 (8)
O2	0.0305 (11)	0.0285 (10)	0.0402 (11)	0.0053 (8)	0.0078 (9)	0.0010 (8)
O3	0.0349 (12)	0.0293 (11)	0.0444 (13)	0.0026 (8)	−0.0129 (10)	−0.0053 (8)
O4	0.0356 (12)	0.0224 (9)	0.0354 (11)	0.0087 (8)	−0.0053 (8)	0.0010 (8)

### Geometric parameters (Å, º)

**Table:**

N1—C1	1.493 (3)	C3—H3A	0.9900
N1—H1N1	0.9100	C3—H3B	0.9900
N1—H2N1	0.9100	C4—C5	1.506 (5)
N1—H3N1	0.9100	C4—H4A	0.9900
N2—C2	1.492 (3)	C4—H4B	0.9900
N2—H1N2	0.9100	C5—C6	1.523 (4)
N2—H2N2	0.9100	C5—H5A	0.9900
N2—H3N2	0.9100	C5—H5B	0.9900
C1—C2	1.514 (4)	C6—H6A	0.9900
C1—C6	1.525 (4)	C6—H6B	0.9900
C1—H1	1.0000	Cr1—O3	1.628 (2)
C2—C3	1.525 (4)	Cr1—O2	1.6394 (19)
C2—H2	1.0000	Cr1—O1	1.6584 (19)
C3—C4	1.520 (4)	Cr1—O4	1.6654 (19)
			
C1—N1—H1N1	109.5	C4—C3—H3B	109.2
C1—N1—H2N1	109.5	C2—C3—H3B	109.2
H1N1—N1—H2N1	109.5	H3A—C3—H3B	107.9
C1—N1—H3N1	109.5	C5—C4—C3	111.0 (3)
H1N1—N1—H3N1	109.5	C5—C4—H4A	109.4
H2N1—N1—H3N1	109.5	C3—C4—H4A	109.4
C2—N2—H1N2	109.5	C5—C4—H4B	109.4
C2—N2—H2N2	109.5	C3—C4—H4B	109.4
H1N2—N2—H2N2	109.5	H4A—C4—H4B	108.0
C2—N2—H3N2	109.5	C4—C5—C6	111.3 (2)
H1N2—N2—H3N2	109.5	C4—C5—H5A	109.4
H2N2—N2—H3N2	109.5	C6—C5—H5A	109.4
N1—C1—C2	113.7 (2)	C4—C5—H5B	109.4
N1—C1—C6	109.5 (2)	C6—C5—H5B	109.4
C2—C1—C6	109.2 (2)	H5A—C5—H5B	108.0
N1—C1—H1	108.1	C5—C6—C1	111.1 (2)
C2—C1—H1	108.1	C5—C6—H6A	109.4
C6—C1—H1	108.1	C1—C6—H6A	109.4
N2—C2—C1	112.8 (2)	C5—C6—H6B	109.4
N2—C2—C3	108.3 (2)	C1—C6—H6B	109.4
C1—C2—C3	109.7 (2)	H6A—C6—H6B	108.0
N2—C2—H2	108.7	O3—Cr1—O2	108.60 (11)
C1—C2—H2	108.7	O3—Cr1—O1	111.43 (11)
C3—C2—H2	108.7	O2—Cr1—O1	109.72 (10)
C4—C3—C2	112.0 (3)	O3—Cr1—O4	109.76 (10)
C4—C3—H3A	109.2	O2—Cr1—O4	108.30 (10)
C2—C3—H3A	109.2	O1—Cr1—O4	108.97 (10)
			
N1—C1—C2—N2	−57.5 (3)	C2—C3—C4—C5	54.6 (4)
C6—C1—C2—N2	179.8 (2)	C3—C4—C5—C6	−53.4 (4)
N1—C1—C2—C3	−178.3 (2)	C4—C5—C6—C1	56.5 (3)
C6—C1—C2—C3	59.0 (3)	N1—C1—C6—C5	175.7 (2)
N2—C2—C3—C4	178.8 (3)	C2—C1—C6—C5	−59.1 (3)
C1—C2—C3—C4	−57.6 (3)		

### Hydrogen-bond geometry (Å, º)

**Table:**

*D*—H···*A*	*D*—H	H···*A*	*D*···*A*	*D*—H···*A*
N1—H1*N*1···O2^i^	0.91	1.99	2.896 (3)	172
N1—H3*N*1···O1^ii^	0.91	2.00	2.884 (3)	164
N1—H2*N*1···O4	0.91	1.81	2.713 (3)	175
N2—H1*N*2···O4^i^	0.91	1.87	2.771 (3)	169
N2—H3*N*2···O2^iii^	0.91	2.56	3.104 (3)	119
N2—H3*N*2···O3^iii^	0.91	2.04	2.927 (3)	166
N2—H2*N*2···O1^iv^	0.91	1.86	2.748 (3)	165

Symmetry codes: (i) −*x*+1, −*y*+1, −*z*+1; (ii) −*x*+3/2, *y*−1/2, *z*; (iii) −*x*+1/2, *y*−1/2, *z*; (iv) *x*, *y*−1, *z*.
